# Supporting data for impact of filler composition on mechanical and dynamic response of 3-D printed silicone-based nanocomposite elastomers

**DOI:** 10.1016/j.dib.2020.106240

**Published:** 2020-09-01

**Authors:** Samantha J. Talley, Brittany Branch, Cynthia F. Welch, Chi Hoon Park, Dana M. Dattelbaum, Kwan-Soo Lee

**Affiliations:** a Los Alamos National Laboratory, Los Alamos, NM 87545, United States; b Sandia National Laboratory, Albuquerque, NM 87185, United States; c Department of Energy Engineering, Gyeongnam National University of Science and Technology (GNTECH), Jinju-si 660-758, Republic of Korea

**Keywords:** 3-D printing, Nanocomposite elastomer, Molecular dynamics simulation, Dynamic response, Silicone

## Abstract

This research reports on the physical and mechanical effects of various filler materials used in direct ink write (DIW) 3-D printing resins. The data reported herein supports interpretation and discussion provided in the research article “Impact of Filler Composition on Mechanical and Dynamic Response of 3-D Printed Silicone-based Nanocomposite Elastomers” [1]. The datasheet describes the model structures and the interaction energies between the fillers and the other components by using Molecular Dynamics (MD) simulations. This report includes mechanical responses of single-cubic (SC) and face-centered tetragonal (FCT) structures printed using new DIW resin formulations (polydimethylsiloxane-based silicones filled with aluminum oxide, graphite, or titanium dioxide). Using MD simulations and mechanical data, the overall flexibility and interactions between resin components are fully characterized.

## Specifications Table

**Table:** 

Subject	Materials Science
Specific subject area	Preparation and characterization of 3-D printed silicone-based nanocomposite elastomers
Type of data	Image, Table, and Figure
How data were acquired	Molecular dynamics simulations, Engineering stress-strain compression data, Storage and loss moduli and tan δ at low compressive strains as a function of oscillatory frequency
Data format	Raw and analyzed
Parameters for data collection	We described “Parameters for data collection” in Experimental Design, Materials, and Methods section.
Description of data collection	We described “Parameters for data collection” in Experimental Design, Materials, and Methods section.
Data source location	Los Alamos, New Mexico, United States of America
Data accessibility	All data is accessible within this article
Related research article	Samantha J. Talley, Brittany Branch, Cynthia F. Welch, Chi Hoon Park, John Watt, Lindsey Kuettner, Brian Patterson, Dana M. Dattelbaum, and Kwan-Soo Lee, Impact of Filler Composition on Mechanical and Dynamic Response of 3-D Printed Silicone-based Nanocomposite Elastomers, Composites Science and Technology, submitted [1]

## Value of the Data

• The data shows the mechanical responses of simple cubic (SC) and face-centered tetragonal (FCT) pads which are composed of polydimethylsiloxane-based polymers filled with aluminum oxide, graphite, or titanium dioxide.
• This data can benefit researchers of materials in the field of formulation chemistry, polymer process engineering, additive manufacturing, and molecular dynamics simulation.
• Guidelines for the mechanical characterization of hybrid composite materials are provided.

## 1 Data Description

**Fig. 1:**
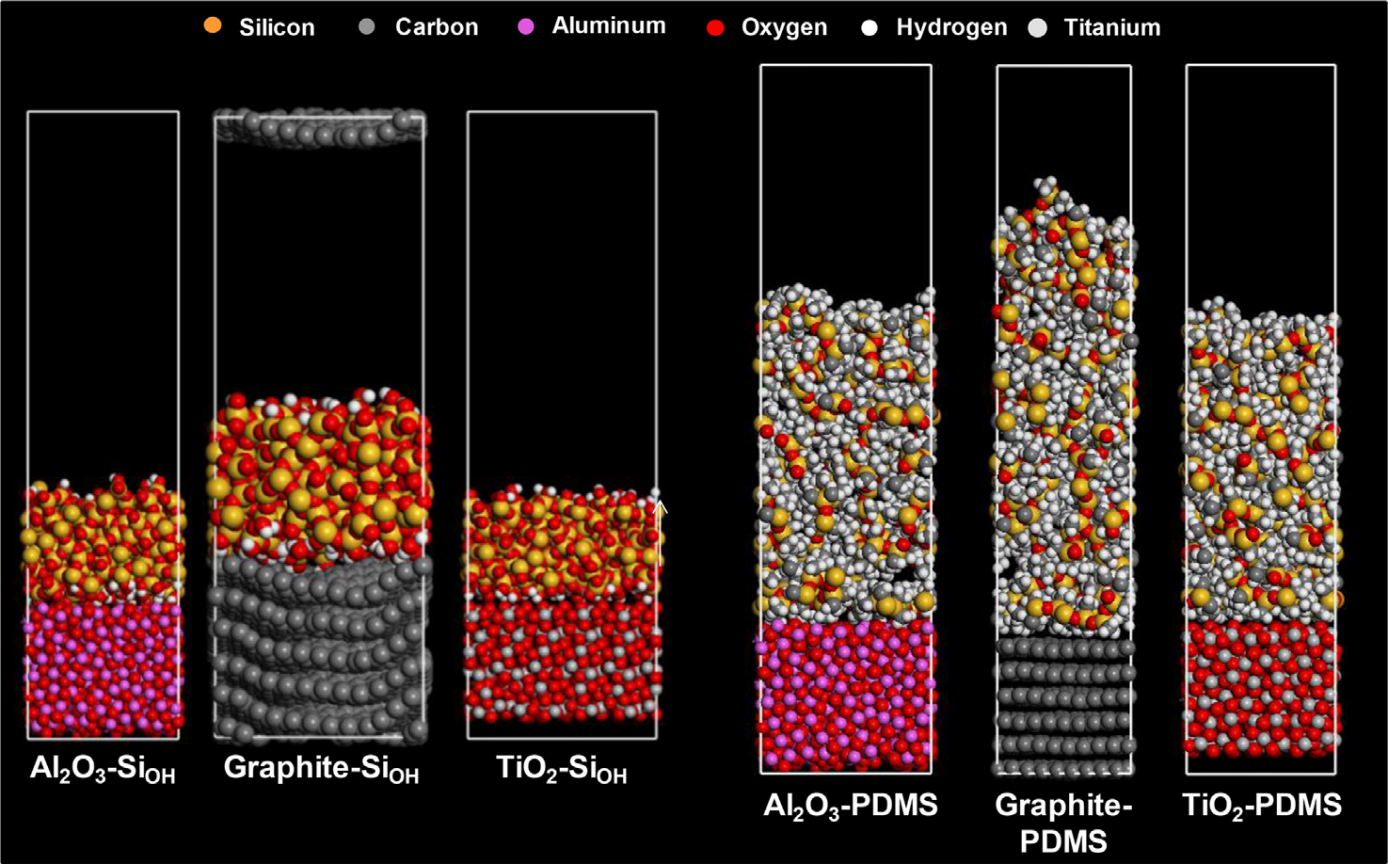
Model structures for Molecular Dynamics (MD) simulations.

This work provides the model structures acquired in molecular dynamics (MD) simulations to calculate the molecular interactions between the components in the composite resins, so that the two-layered models were built and equilibrated (Fig. 1 and Table 1), as well as the mechanical responses of the simple cubic (SC) and face-centered tetragonal (FCT) having three different formulations. (Figs. 4–8.) [1]. Six different model systems are explored by MD simulations: a) Al_2_O_3_-Si_OH_ (fumed silica; non-treated silica), graphite-Si_OH_, TiO_2_-Si_OH_, Al_2_O_3_-PDMS (PDMS; polydimethylsiloxane), graphite-PDMS, and TiO_2_-PDMS (Fig. 1 and Table 1).

**Fig. 2:**
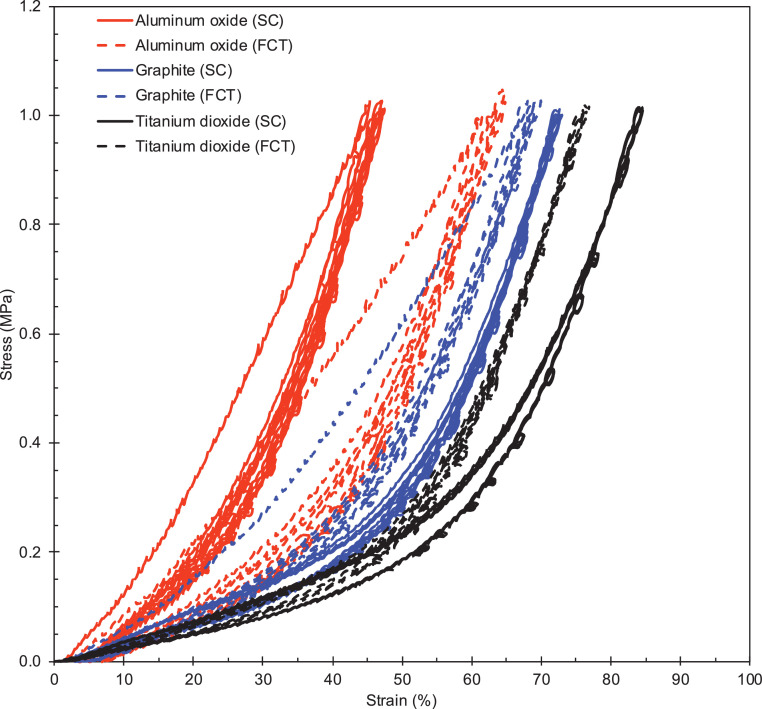
Engineering stress as a function of the engineering strain for SC (solid lines) and FCT (dashed lines) DIW pads obtained at room temperature. Pads are filled with 25 wt.% aluminum oxide (red), 25 wt.% graphite (blue), and 25 wt.% titanium dioxide (black). All four loading cycles are represented. (For interpretation of the references to color in this figure legend, the reader is referred to the web version of this article.)

**Fig. 3:**
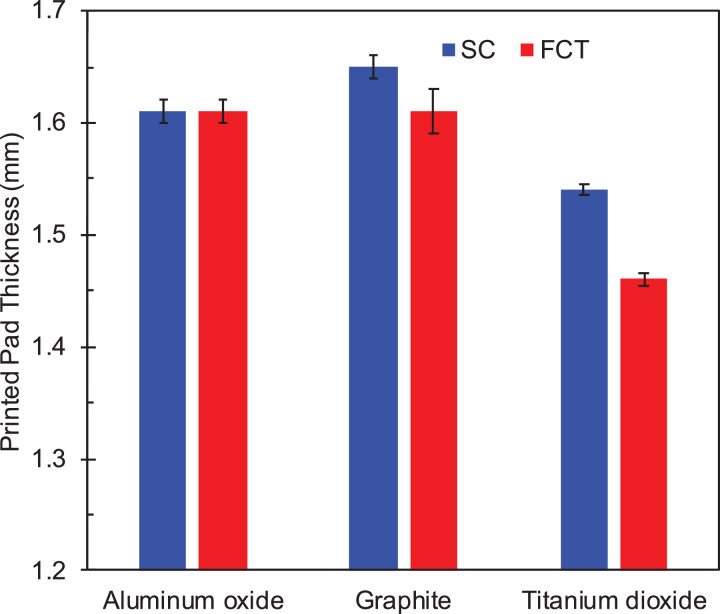
Thickness in mm of 7-layer DIW printed pads where simple cubic (SC) are shown in blue, and face-centered tetragonal (FCT) are shown in red. (For interpretation of the references to color in this figure legend, the reader is referred to the web version of this article.)

**Table 1:** The interaction energies between the fillers and the other components calculated by molecular dynamics (MD) simulations. To calculate the interaction energy per gram, the densities (Al_2_O_3_/Graphite/TiO_2_ = 3.2/2.2/4.23 g/cc) and the primary particle sizes were used.

Model	Interaction Energy (kcal/mol)	Interaction Energy per area (kcal/mol Å^2^)	Interaction Energy per a particle (kcal/mol•ea)	Interaction Energy per gram (kcal/mol•g)
Al_2_O_3_-Si_OH_	−474.7	−0.8771	−4.515 * 10^4^	−1.285 * 10^25^
Al_2_O_3_-PDMS	−453.0	−0.8371	−4.309 * 10^4^	−1.226 * 10^25^
Graphite-Si_OH_	−194.1	−0.2536	−3.187 * 10^4^	−3.458 * 10^24^
Graphite-PDMS	−132.8	−0.2808	−3.528 * 10^4^	−3.829 * 10^24^
TiO_2_-Si_OH_	−326.8	−0.3425	−6.512 * 10^4^	−1.975 * 10^24^
TiO_2_-PDMS	−202.0	−0.3733	−7.096 * 10^4^	−2.152 * 10^24^

**Fig. 4:**
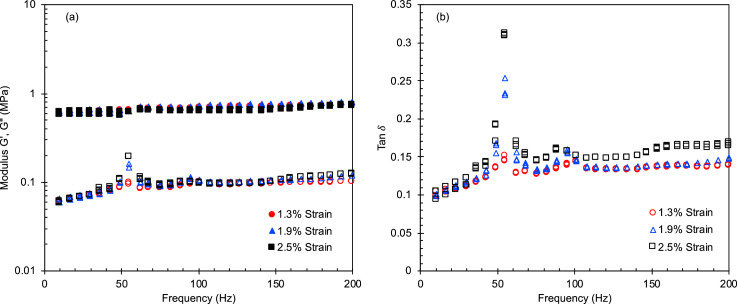
(a) Storage and loss moduli and (b) tan δ at low compressive strains as a function of oscillatory frequency for Al/PDMS SC pads. The data for Al/PDMS FCT pads is described in reference [1].

**Fig. 5:**
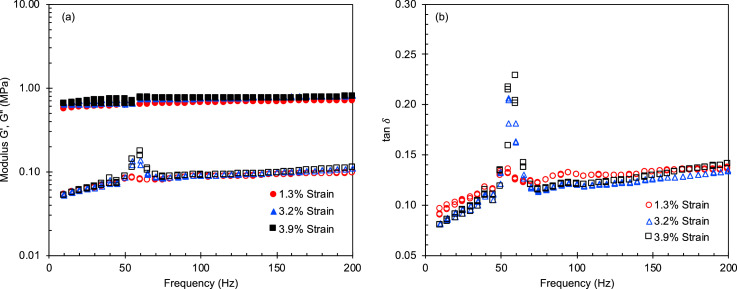
(a) Storage and loss moduli and (b) tan δ at low compressive strains as a function of oscillatory frequency for G/PDMS FCT pads.

**Fig. 6:**
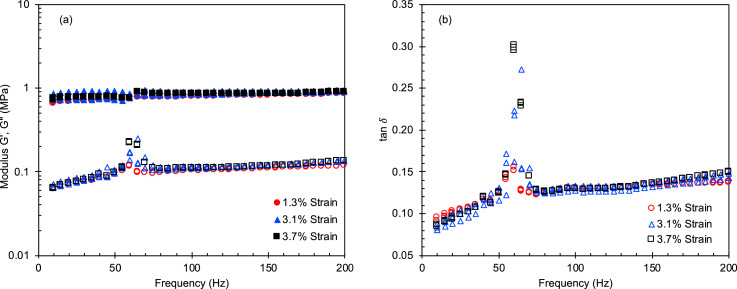
(a) Storage and loss moduli and (b) tan δ at low compressive strains as a function of oscillatory frequency for G/PDMS SC pads.

**Fig. 7:**
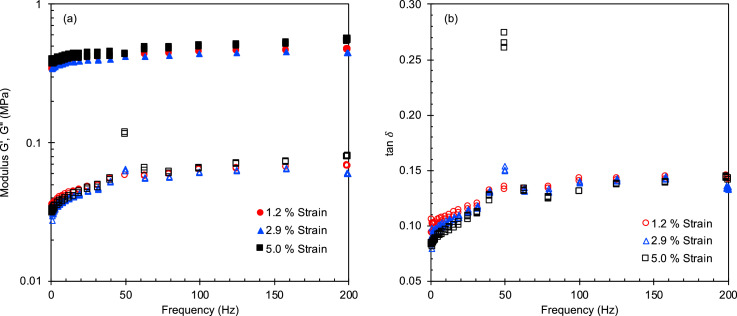
(a) Storage and loss moduli and (b) tan δ at low compressive strains as a function of oscillatory frequency for Ti/PDMS FCT pads.

**Fig. 8:**
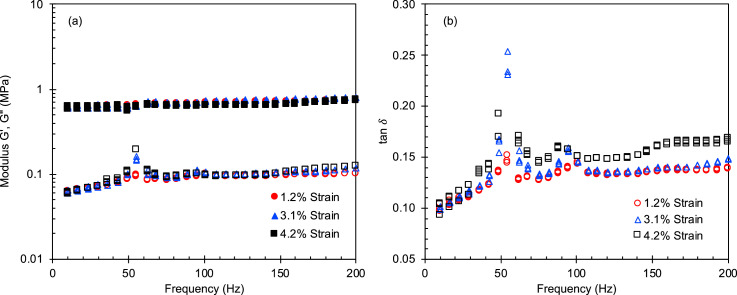
(a) Storage and loss moduli and (b) tan δ at low compressive strains as a function of oscillatory frequency for Ti/PDMS SC pads.

## 2 Experimental Design, Materials, and Methods

### 2.1 Composite resins preparation

Three resins were specifically formulated for direct ink writing (DIW) printing [2]. These resins were composed of 65 wt% of polydimethylsiloxane (PDMS), 10 wt% of fumed silica, and 25 wt% of graphite (Alfa Aesar), TiO_2_ (Evonik Industries), or Al_2_O_3_ (Evonik Industries).

### 2.2 Molecular dynamics (MD) simulations

Amorphous Cell module was used as a model builder. The models were geometrically optimized until their energies were stable, and were equilibrated by MD simulation with NVT (constant number of atoms, volume, and temperature) ensemble, in which the temperature was slowly increased from 0 K to 298 K in stepwise to avoid the calculation failure. The final production run for the interaction energy calculation was performed with NVT ensemble at 298 K and for 100 ps. In this simulation, we used Materials studio program package (BIOVIA Software Inc., CA, USA) and COMPASS II (Condensed-phase Optimized Molecular Potentials for Atomistic Simulation Studies II) force field were used as a force-field and force-field types and charges of all atoms were set to the default values [3], [4], [5]. Ewald and atom based summation method were used for electrostatic and van der Waals interactions, respectively.

### 2.3 Uniaxial compression and dynamic mechanical analysis

The compression test was performed using an ADMET eXpert 7601 testing system. Samples with dimensions of 2 × 2 cm were compressed for 4 cycles to a maximum stress of 1.0 MPa at a strain rate of 0.5%/sec. Dynamic mechanical analysis was performed in compression mode with a TA Instruments Q800 Dynamic Mechanical Analyzer (DMA), using 15-mm compression plates at ambient temperature (∼23 °C). Oscillatory strain sweeps were conducted at a frequency of 1 Hz to determine the linear viscoelastic regime for each sample. Subsequently, oscillatory frequency sweeps from 1 to 200 Hz were performed at strains within this regime; three cycles of each frequency sweep confirmed reproducibility.

## Declaration of Competing Interest

The authors declare that they have no known competing financial interests or personal relationships which have, or could be perceived to have, influenced the work reported in this article.

## Appendix Supplementary materials

Image, application 1
